# Value of an automated machine learning model with post-hoc explanation for predicting healthcare-seeking delays among residents in Tibetan regions

**DOI:** 10.3389/fpubh.2026.1682879

**Published:** 2026-02-17

**Authors:** Zhenzhong Xi, Chenxing Meng, Qian Li, Yisha Xu, Peng Wu, Zhigang Zhang, Tingyong Han, Liangjie Zhang, Xinxuan Han

**Affiliations:** 1 The 945th Hospital of the Joint Logistics Support Force, PLA, Ya’an, Sichuan, China; 2 General Hospital of Western Theater Command, PLA, Chengdu, Sichuan, China; 3 Ya’an People’s Hospital, Ya’an, Sichuan, China; 4 Yucheng District People’s Hospital of Ya’an, Ya’an, Sichuan, China; 5 Mingshan District People’s Hospital of Ya’an, Ya’an, Sichuan, China; 6 Affiliated Hospital of Ya’an Polytechnic College, Ya’an, Sichuan, China; 7 Ya’an Hospital of Traditional Chinese Medicine, Ya’an, Sichuan, China

**Keywords:** automated machine learning, clinical decision support system, healthcare-seeking delay, interpretability analysis, Tibetan healthcare

## Abstract

**Objective:**

This study aimed to investigate key determinants of healthcare-seeking delays among Tibetan residents and develop predictive models using automated machine learning (AutoML) with post-hoc SHAP interpretation alongside a clinical decision support system.

**Methods:**

Face-to-face surveys using structured questionnaires were administered to 1,879 Tibetan residents. Data processing employed an AutoML framework: datasets were partitioned into training (*n* = 1,503) and testing (*n* = 376) subsets at an 8:2 ratio. Standardized preprocessing—including outlier rectification, one-hot encoding (OHE), and random forest-based multiple imputation (MI)—was applied. Model validation integrated 5-fold cross-validation and SHapley Additive exPlanations (SHAP) analysis.

**Results:**

Among 1,879 participants, the healthcare-seeking delay incidence was 41.99%. The LightGBM model significantly outperformed conventional approaches (AUC > 0.86). SHAP feature importance analysis revealed the predictor hierarchy: Age > County hospital quality score > Distance to county hospital > Township health center quality score > Able to communicate in Chinese.

**Conclusion:**

A high-performance model with post-hoc SHAP interpretation accurately identifies geographical, cultural, and healthcare resource variables to accurately identify high-risk populations. The developed clinical decision support system enables risk computation through modular interfaces, providing an evidence-based tool for optimizing hierarchical diagnosis and resource allocation in Tibetan healthcare.

## 1 Introduction

The persistent issue of delayed medical care-seeking among residents in Tibetan regions, including Tibet, has emerged as a critical challenge in China’s ethnic healthcare services. Constrained by geographic barriers, cultural practices, and socioeconomic factors, local populations often adopt a “neglecting minor ailments, enduring severe illnesses” approach to healthcare (1–3). Recent epidemiological surveys reveal a median interval of 17.3 days between symptom recognition and initial medical consultation among Tibetan agro-pastoralists, significantly exceeding the national median of 5.2 days (4). Such delays not only elevate risks of disease progression but also exacerbate healthcare resource inefficiencies and public health burdens.

Conventional predictive analyses, predominantly reliant on traditional statistical methods like logistic regression, demonstrate strong interpretability yet fail to address the distinct complexities of healthcare data from Tibetan communities (5). These datasets exhibit characteristics typical of big data—volume, variability, veracity heterogeneity, and velocity—while care-seeking behaviors are jointly influenced by objective determinants (e.g., transportation accessibility, economic capacity) and subjective factors (e.g., ethnic beliefs, health literacy), thereby eluding conventional analytical approaches. Automated Machine Learning (AutoML) offers a promising solution by automating feature engineering, algorithm selection, and hyperparameter optimization to uncover latent patterns from intricate data (6). While machine learning demonstrates remarkable predictive capabilities, its clinical adoption faces inherent limitations. Key among these is the “black-box” dilemma—traditional ML models provide insufficient transparency regarding decision pathways, particularly problematic in healthcare contexts requiring causal attribution (7). Additional constraints include: (1) Vulnerability to data bias propagation, especially when handling heterogeneous ethnic healthcare datasets; (2) Limited generalizability across geographically isolated populations such as high-altitude communities; (3) Resource-intensive customization requirements for specialized clinical scenarios. These fundamental constraints necessitate the development of interpretable modeling frameworks. Beyond the specific Explainable AutoML (XAI) approach utilized in this study, the broader field encompasses diverse methodologies—including rule-based systems, inherently interpretable models, and local approximation techniques—that collectively advance model transparency across healthcare domains. It is within this methodological landscape that XAI technology offers critical solution pathways, reconciling predictive accuracy with clinical interpretability needs through its unique capacity for quantitative assessment of culture-specific variables in complex healthcare environments.

XAI technology enables comprehensive evaluation of multidimensional predictors (e.g., geographical isolation, linguistic barriers, cultural norms) through quantitative assessment of variable-specific contributions to care-seeking delays (8, 9). Crucially, XAI systems visually elucidate culture-mediated variables (e.g., religious practices, traditional remedies), bridging clinician-patient understanding gaps via intuitive graphical interfaces and concise explanatory outputs (10). Moreover, XAI pinpoints actionable determinants (e.g., transportation deficits in remote areas), thereby guiding evidence-based resource allocation such as optimized clinic placement or enhanced patient transfer services (11). This study therefore aims to: (1) evaluate the implementation value of XAI for predicting healthcare-seeking delays in Tibetan populations; (2) construct a multidimensional data-integrated prediction framework; and (3) establish a transparent intelligent decision-support tool addressing limitations of conventional diagnostic paradigms.

## 2 Methods

### 2.1 Study participants

This prospective multi-center cohort study leverages data collected from seven public hospitals frequently accessed by Tibetan patients for cross-regional healthcare services (June 2024–June 2025). Participating institutions include: The 945th Hospital of Joint Logistics Support Force, PLA; General Hospital of Western Theater Command, PLA; Ya’an People’s Hospital; Ya’an Second People’s Hospital; Ya’an Hospital of Traditional Chinese Medicine; Ya’an Mingshan District People’s Hospital; Affiliated Hospital of Ya’an Polytechnic. All enrolled participants provided written informed consent. The study protocol received ethical approval from the Institutional Review Board of the coordinating center (The 945th Hospital of Joint Logistics Support Force, PLA; Approval No.: 2023-10014).

*Inclusion Criteria:* (1) Residents with ≥5 years of continuous residency in Tibetan regions; (2) Ethnicities: Tibetan, Han Chinese, or other ethnic minorities.

*Exclusion Criteria:* Inability to complete surveys due to language or communication barriers.

### 2.2 Data collection

Data were gathered through structured questionnaires administered via face-to-face interviews with Tibetan patients. The following clinical parameters were documented: (1) Demographic variables: Age, gender, education level, occupation; (2) Socioeconomic indicators: Able to communicate in Chinese (Yes/No), financial hardship (Yes/No); (3) Healthcare accessibility: Distance to village clinic (km), township health center (km), and county hospital (km); (4) Clinical characteristics: Chronic disease history (Yes/No), self-rated symptom severity (Yes/No), health center quality rating (0–10 scale), county hospital quality rating (0–10 scale); (5) Symptom domains (Yes/No): Musculoskeletal (e.g., joint pain/stiffness, back pain)/Cardiopulmonary (e.g., palpitations, dyspnea, chest pain, cough)/Gastrointestinal (e.g., nausea, vomiting, diarrhea)/Neurological (e.g., headache, dizziness)/Other (e.g., abnormal vaginal discharge, weight loss).

*Healthcare-Seeking Delay Definition:* Healthcare-Seeking delay was rigorously defined as an interval exceeding 14 days between symptom onset and initial professional medical intervention. This threshold synthesizes multidimensional evidence: Chinese clinical studies established a golden intervention window for acute respiratory diseases (12), while the Health Commission Bulletin of Tibet Autonomous Region (2022) empirically confirmed a mean healthcare-seeking duration of 16.3 days among agricultural-pastoral populations. Collectively, these findings validate the 14-day demarcation as both clinically grounded and regionally pragmatic. The quality control protocol encompassed rigorous multidimensional safeguards. Questionnaire development was anchored in multidisciplinary Delphi consultations (>3 rounds) and systematic literature synthesis, followed by pilot testing in Baoxing County, Sichuan—a socioeconomically comparable non-study region (*n* ≥ 50). Scale reliability was confirmed via Cronbach’s *α* (>0.8), with ambiguous items refined through item analysis. Complementary validity assessment employed exploratory factor analysis (EFA) on pilot data (*n* = 50) to confirm construct validity. Varimax-rotated principal component analysis demonstrated unidimensional factor structures across all domains, with all item loadings exceeding 0.45. Content validity was further established through expert evaluation of item relevance (averaged Content Validity Index, CVI = 0.89) based on Delphi consensus thresholds for medical instruments. Investigators, comprising clinically trained Tibetan-competent staff, underwent comprehensive instruction encompassing theoretical modules (cultural customs, standardized Tibetan medical terminology, item interpretation protocols) and practical simulations for refusal mitigation and emergent scenarios; certification was contingent upon post-training competency assessment. Following daily interviews, a tiered verification framework was implemented: investigators conducted self-reviews, performed peer cross-validation, and submitted finalized questionnaires to the lead researcher for audit. This integrated approach ensured maximal data fidelity throughout the study period.

### 2.3 Model construction

**Figure 1:**
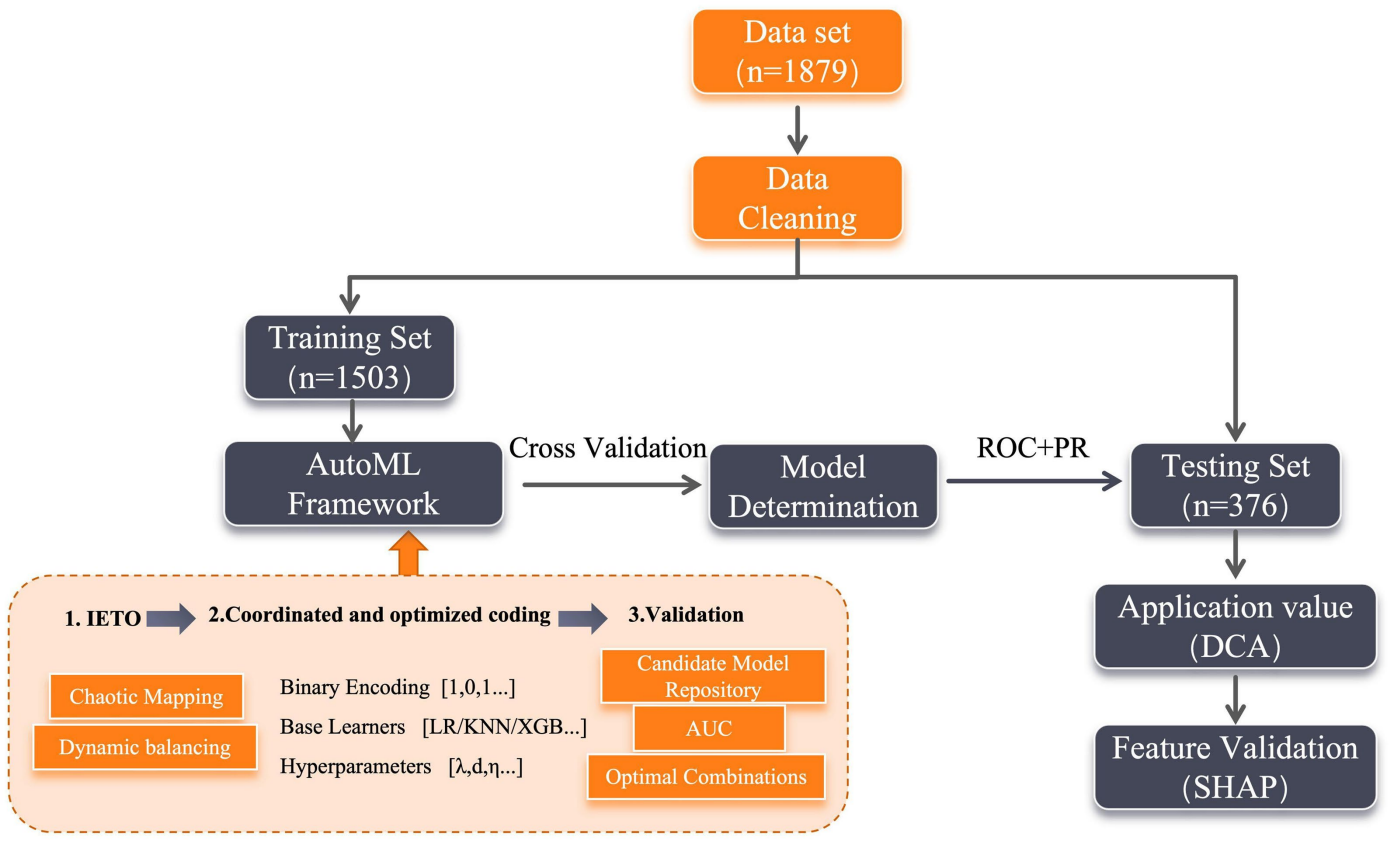
Full workflow.

Using automated machine learning (AutoML) as the core methodological framework, the dataset was partitioned into training (*n* = 1,503) and testing (*n* = 376) subsets at an 8:2 ratio. Both subsets underwent standardized preprocessing, comprising outlier rectification, one-hot encoding (OHE) of categorical variables, and random forest-based multiple imputation (MI) for missing values. The total missing data proportion across all variables was 3.2%, with no single variable exceeding 5% missingness, satisfying the threshold for robust MI application. During model development, the training set was optimized via 5-fold cross-validation, while the testing set quantified the final model’s generalization efficacy. To validate clinical utility, dual corroboration strategies were deployed: Decision Curve Analysis (DCA) delineated benefit thresholds across risk spectra, SHapley Additive exPlanations (SHAP) for interpretability auditing (full workflow: Figure 1). Notably, while the proposed AutoML framework automates model selection and hyperparameter tuning to optimize predictive accuracy, interpretability per se was not incorporated as an optimization objective during the search process. Instead, the explainability component functions as a post-hoc interpretation module applied solely to the final selected model using SHAP.

Core architecture and workflow: The proposed IETO-AutoML framework integrates an Improved Exponential-Trigonometric Optimization (IETO) algorithm as a meta-optimizer within a hierarchical AutoML pipeline (see PSEUDOCODE 1 for the schematic workflow). This integration operates through a sequential-cooperative mechanism: The AutoML pipeline manages the overarching model construction process, including data preprocessing, candidate model instantiation, training, and validation. The IETO algorithm acts as an intelligent search engine that navigates the combined space of feature subsets, base learner choices, and their hyperparameters. It communicates with the AutoML pipeline through a 0–1 encoded solution vector. For each candidate solution (representing a specific feature subset, learner, and hyperparameter set), the IETO requests the AutoML pipeline to construct and evaluate the corresponding model via cross-validation. The performance metric (AUC) is then fed back to IETO to guide its evolutionary search towards optimal configurations.

ALGORITHM 1 Workflow of the IETO-AutoML collaborative optimization framework.
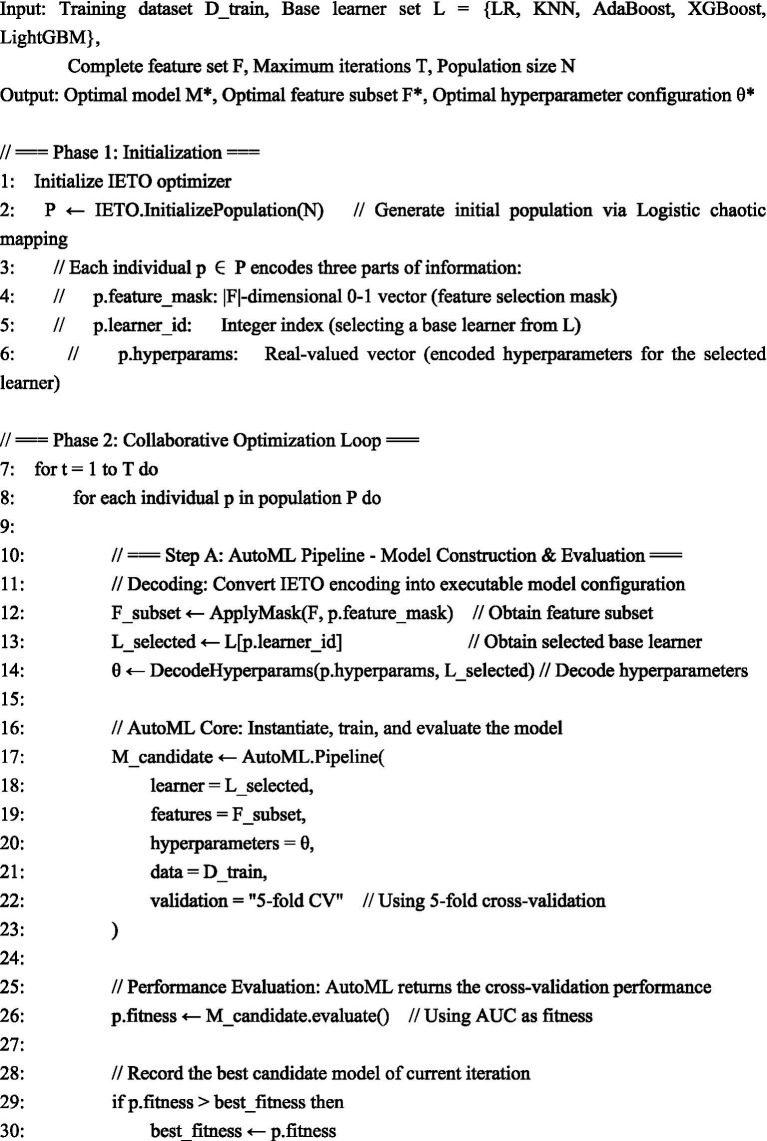

The IETO meta-optimizer: To circumvent premature convergence in conventional methods, the IETO enhanced the foundational Exponential-Trigonometric Optimization (ETO) through three innovations (13): (1) Population initialization via Logistic chaotic mapping to ensure high entropy and diversity; (2) A balanced search strategy combining spiral exploration and Cauchy mutation perturbation for dynamic space traversal; (3) An adaptive mechanism to trade off between local refinement and global discovery. Within our framework, the IETO iteratively generates binary-coded vectors. The first segment encodes the feature subset (1 for selected, 0 for excluded), the second segment selects a base learner from the predefined pool {Logistic Regression (LR), K-Nearest Neighbors (KNN), AdaBoost, XGBoost, LightGBM}, and the subsequent segments encode the hyperparameters for the selected learner (see Table 1 for configurations). The algorithm’s search objective is to maximize the 5-fold cross-validation AUC on the training set. The robustness of IETO as an optimizer was preliminarily verified against the CEC2022 benchmark suite (14), where it demonstrated superior convergence and solution quality compared to native ETO, Whale Optimization Algorithm (WOA), and Particle Swarm Optimization (PSO).

**Table 1:** Hyperparameter search space and optimized configurations for base learners in IETO-AutoML.

Base Learner	Hyperparameter search space	Rationale for search space
Logistic regression (LR)	C: [1e-3, 1e3] (log), penalty: [‘l1’, ‘l2’]	‘C’ controls regularization strength; ‘penalty’ selects norm to prevent overfitting.
K-nearest neighbors (KNN)	n_neighbors: [3, 15], weights: [‘uniform’, ‘distance’]	Balances bias-variance trade-off; distance weighting gives more influence to closer neighbors.
AdaBoost	n_estimators: [50, 200], learning_rate: [0.01, 1.0]	Number of sequential weak learners and their contribution rate; key for boosting performance.
XGBoost	max_depth: [3, 10], learning_rate: [0.01, 0.3], n_estimators: [100, 300]	Controls tree complexity, step size shrinkage, and number of boosting rounds to avoid overfitting.
LightGBM	num_leaves: [31, 127], learning_rate: [0.01, 0.3], feature_fraction: [0.7, 1.0]	Leaf-wise growth control, learning speed, and stochastic feature selection for efficiency & generalization.

The search was conducted in continuous/discrete spaces as appropriate.; Specific values may vary per run but fall within the defined spaces.

Feature selection mechanism: Variable selection is an embedded and optimized component within the IETO-AutoML search process. The elimination principle is driven by performance. The IETO algorithm evaluates the contribution of a variable subset holistically within the context of a specific model and its hyperparameters. A variable is effectively “eliminated” (assigned ‘0’ in the encoding) if its inclusion, in combination with the current model configuration, fails to improve the cross-validation AUC compared to alternative subsets. This process is not based on a univariate statistical threshold but on the variable’s utility within the multivariate predictive ensemble. The final model selected the five most important variables because that particular subset, combined with the optimal learner (LightGBM) and its tuned hyperparameters, yielded the highest AUC. The specific hyperparameter configurations for each base learner, optimized within plausible ranges by IETO, are justified in Table 1.

### 2.4 Evaluation metrics

A comprehensive suite of metrics was employed to holistically assess classification performance and clinical applicability. The foundational indicators encompassed Precision (PRE) to quantify positive predictive accuracy, Sensitivity (SEN) for true positive recognition capacity, Specificity (SPE) reflecting true negative exclusion efficacy, and Accuracy (ACC) representing overall classification validity; F1-score balanced the inherent trade-off between PRE and SEN; ROC-AUC measured discriminatory power across latent categories, while PR-AUC evaluated robustness under class-imbalanced scenarios. For clinical utility appraisal, Decision Curve Analysis (DCA) dynamically compared net benefit differentials against baseline intervention strategies (treat-all or treat-none) across risk thresholds, thereby validating operational validity domains and risk-assessment generalizability.

### 2.5 Interpretability analysis

As a post-hoc interpretation approach independent of the AutoML optimization, SHAP was applied to the finalized model to ensure biological plausibility and statistical reliability of the model interpretation, while significantly enhancing its clinical trustworthiness and acceptability, the deconstruction of model logic was rigorously executed using the SHAP framework rooted in coalitional game theory. This approach encompassed multiple analytical dimensions: Shapley values numerically attributed feature contribution weights to individual predictions; summary plots elucidated global feature importance hierarchies; dependence plots delineated nonlinear feature-outcome interactions; waterfall plots visualized prediction-specific pathways; force plots decomposed decision logic across feature dimensions; and decision plot matrices traced multidimensional reasoning sequences. Furthermore, interaction plots were incorporated to dissect synergistic effects between features. To fortify methodological robustness and ensure cross-validation consistency, SHAP-derived outputs underwent systematic benchmarking against both machine learning model-intrinsic feature importance rankings and LASSO regression coefficient estimates. This comprehensive methodology guaranteed statistically validated interpretations while maintaining clinically actionable transparency throughout the analytical continuum.

### 2.6 Clinical decision support system

A clinical decision support platform was developed using MATLAB’s App Designer to provide an intelligent prognostic tool. The system integrates a computational core for risk prediction with modular user interfaces that support patient data entry, automated calculation of risk probabilities, and flexible deployment across web-based and local server environments. Interactive graphical components dynamically visualize results, streamlining human-computer interaction and facilitating clinical interpretation. This platform establishes an accessible and scalable infrastructure for precision medicine, designed to assist clinicians in personalized prognosis evaluation.

### 2.7 Statistical analysis

Data underwent standardized processing in SPSS 26.0: continuous variables confirming normal distribution were expressed as mean ± standard deviation, and unordered categorical variables as proportions (%). Intergroup differences were assessed using independent samples *t*-tests (continuous variables) or χ^2^ tests (categorical variables), with statistical significance defined at *p* < 0.05.

## 3 Results

### 3.1 Cohort characteristics

This prospective cohort comprised 1,879 enrolled patients, with delayed healthcare-seeking identified in 789 individuals (41.99%). Ages ranged from 25 to 72 years (mean = 48.38 ± 11.45 years). The cohort was partitioned into training (*n* = 1,503) and testing (*n* = 376) subsets at an 8:2 ratio. Comparative analysis demonstrated no statistically significant differences in baseline characteristics between datasets across all variables (*p* > 0.05), as detailed in Table 2.

**Table 2:** Comparative Analysis of Baseline Characteristics.

Characteristics	Training set (*n* = 1,503)	Testing set (*n* = 376)	*t*/*χ*^2^	*p*-value
Age (years, mean ± SD)	48.52 ± 11.32	47.81 ± 12.91	1.057	0.291
Gender [*n*(%)]			0.977	0.323
Male	654 (43.51)	153 (40.69)		
Female	849 (56.49)	223 (59.31)		
Education level [*n*(%)]			0.011	0.917
Primary school or below	592 (39.39)	147 (39.10)		
Above primary school	911 (60.61)	229 (60.90)		
Occupation [*n*(%)]			2.652	0.103
Farming	718 (47.77)	162 (43.09)		
Non-farming	785 (52.23)	214 (56.91)		
Able to communicate in Chinese [*n*(%)]			0.293	0.588
Yes	1,127 (74.98)	287 (76.33)		
No	376 (25.02)	89 (23.67)		
Family financial difficulty [*n*(%)]			2.682	0.101
Yes	653 (43.45)	181 (48.14)		
No	850 (56.55)	195 (51.86)		
Distance to village clinic (km, mean ± SD)	1.06 ± 0.25	1.09 ± 0.33	1.942	0.052
Distance to township health center (km, mean ± SD)	4.03 ± 1.23	4.15 ± 1.17	1.708	0.088
Distance to county hospital (km, mean ± SD)	21.62 ± 5.87	21.53 ± 6.01	0.265	0.791
Chronic disease history [*n*(%)]			0.348	0.555
Yes	706 (46.97)	183 (48.67)		
No	797 (53.03)	193 (51.33)		
Self-rated symptom severity [*n*(%)]			0.016	0.899
Yes	613 (40.79)	152 (40.43)		
No	890 (59.21)	224 (59.57)		
Township health center quality score (points, mean ± SD)	8.18 ± 2.45	7.95 ± 2.06	1.678	0.094
County hospital quality score (points, mean ± SD)	9.13 ± 3.01	9.28 ± 2.95	0.868	0.386
Musculoskeletal symptoms [*n*(%)]			0.107	0.744
Yes	265 (17.63)	69 (18.35)		
No	1,238 (82.37)	307 (81.65)		
Cardiopulmonary symptoms [*n*(%)]			0.185	0.667
Yes	506 (33.67)	131 (34.84)		
No	997 (66.33)	245 (65.16)		
Gastrointestinal symptoms [*n*(%)]			0.848	0.357
Yes	246 (16.37)	69 (18.35)		
No	1,257 (83.63)	307 (81.65)		
Neurological symptoms [*n*(%)]			0.010	0.920
Yes	189 (12.57)	48 (12.77)		
No	1,314 (87.43)	328 (87.23)		

### 3.2 Performance assessment of algorithmic refinement

**Figure 2:**
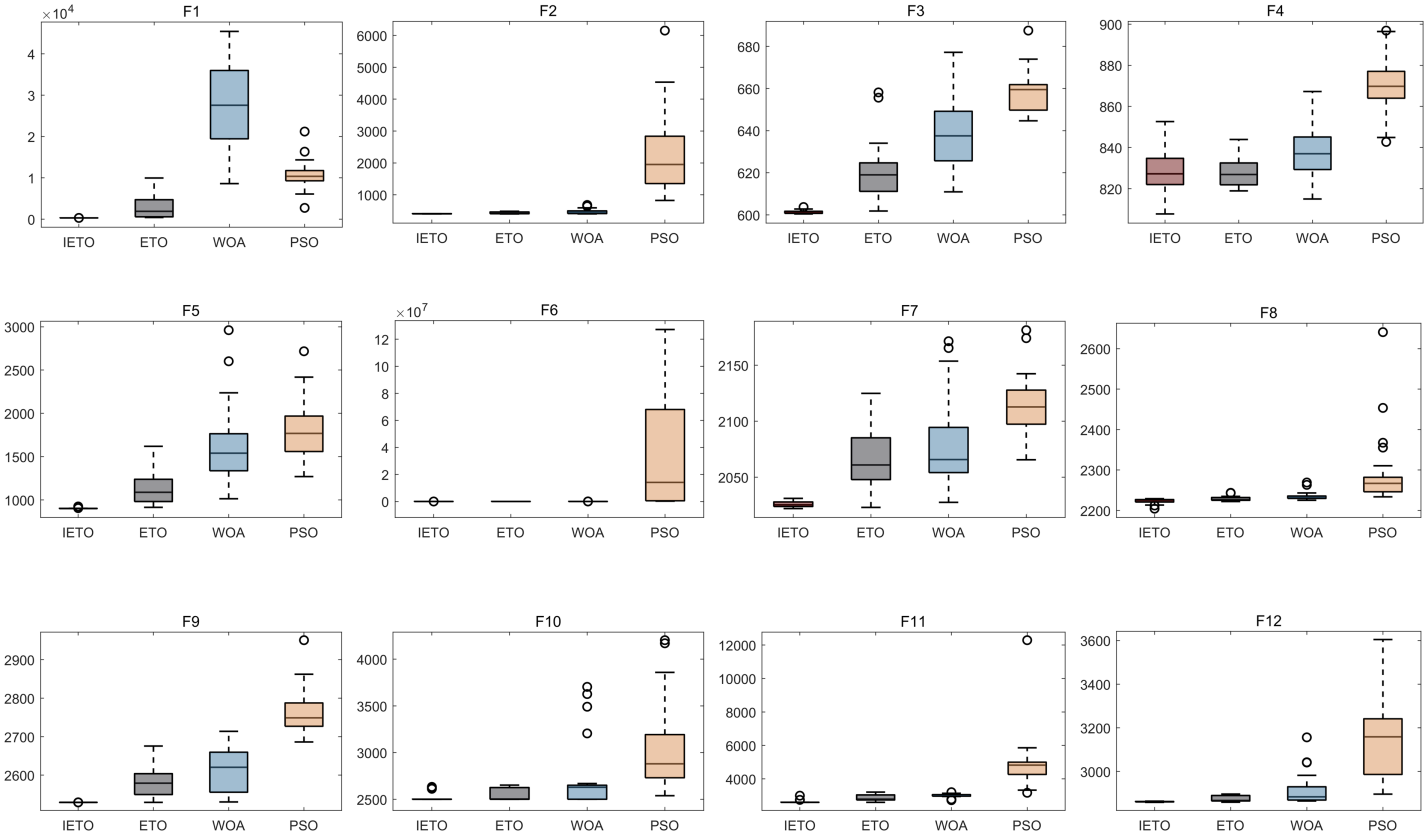
Comparative evaluation of optimization efficacy in swarm intelligence algorithms. The horizontal axis denotes different CEC2022 benchmark functions (or function indices), while the vertical axis represents the best fitness values (objective function values) obtained over 30 independent algorithm executions. Lower values indicate superior optimization efficacy. Each box plot illustrates the statistical distribution of 30 runs for a given algorithm on a specific test function. Narrower interquartile ranges (IQR) and whisker spans (extending to ±1.5 × IQR) reflect enhanced algorithmic stability. As visually evident, IETO exhibits markedly more compact distributions (lower median positions and reduced IQRs) than ETO, WOA, and PSO across most functions, validating its superior solution quality and robust convergence behavior.

**Figure 3:**
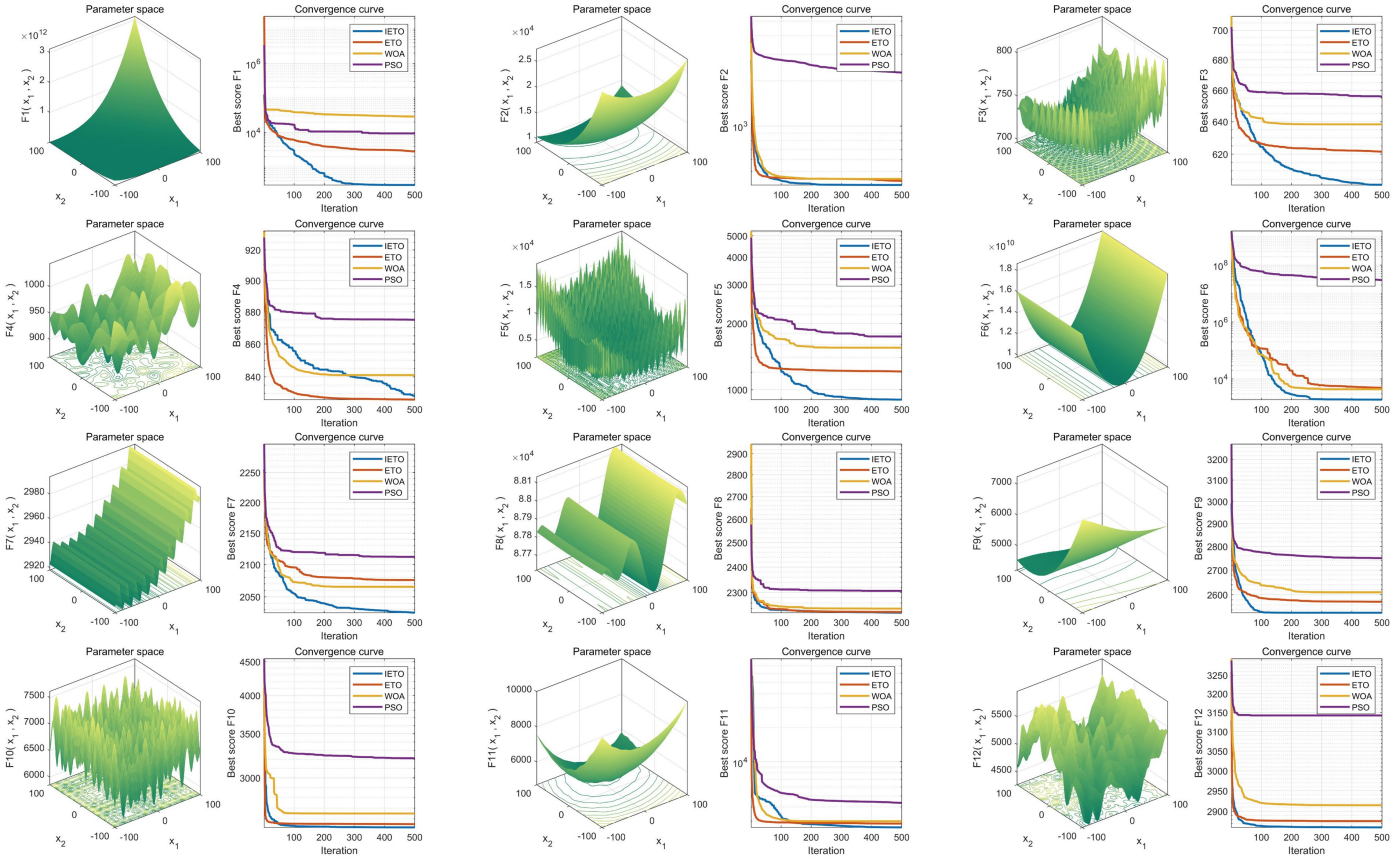
Comparative analysis of convergence dynamics in swarm intelligence algorithms. The horizontal axis indicates iteration counts (1–500), and the vertical axis quantifies either the contemporary population mean fitness or best fitness values. Lower values denote higher solution quality. Each trajectory depicts evolutionary fitness progression for one algorithm. Accelerated initial descent rates characterize rapid convergence, while sustained declines toward lower asymptotic plateaus signify enhanced capability for escaping local optima. The IETO trajectory (distinguished by [specified line style/color]) demonstrates the steepest initial convergence gradient and achieves the lowest terminal values across functions, confirming its dual proficiency in swift convergence and global exploration efficacy.

To validate the optimization efficacy of the enhanced IETO algorithm, this investigation performed comparative evaluations against the original ETO, WOA, and PSO algorithms. Experiments utilized the full suite of 12 benchmark functions from the CEC2022 test set, with all functions exhibiting a dimensionality of 10, a population size of 30, and a maximum iteration threshold of 500. Independent executions were repeated 30 instances to ensure statistical robustness. Through correlation of the 30 cumulative outcomes, box plots were generated to appraise optimization stability across algorithms. Results demonstrated that IETO manifested exceptional proficiency in the predominant majority of test functions, exhibiting markedly superior stability relative to the original ETO and peer comparative algorithms (Figure 2). Subsequent examination of convergence trajectories revealed IETO possessed accelerated convergence velocity and the minimal propensity for entrapment in local optima during iterative sequences (Figure 3). Collectively, these experimental observations unequivocally substantiate the IETO algorithm’s pronounced advantages in global optimization capability and convergence efficiency.

### 3.3 Model training performance

**Figure 4:**
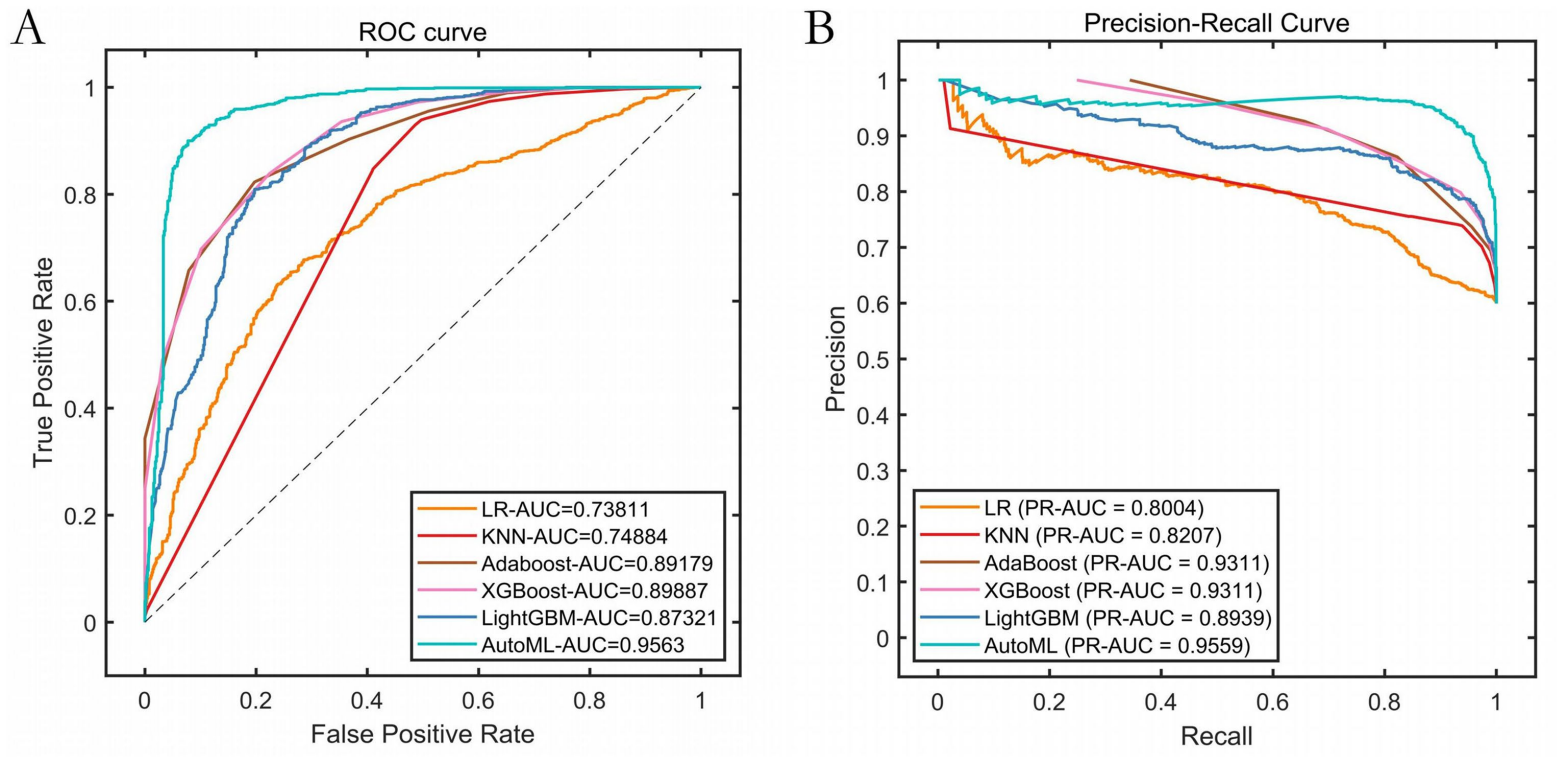
Cross-validation performance of the training set. **(A)** ROC curve of training set; **(B)** PR curve of training set.

Leveraging the feature selection outcomes from the IETO-AutoML framework, five pivotal predictors were definitively identified: age; county hospital quality score; distance to county hospital; community clinic quality score; able to communicate in Chinese. Through the automated optimization architecture, LightGBM was autonomously designated as the optimal base learner. Comparative analysis against five conventional models employing cross-validation revealed IETO-AutoML’s substantial superiority in comprehensive performance. It demonstrated remarkable metrics including an F1-index of 0.9243, ROC-AUC of 0.9563, and PR-AUC of 0.9559 (Table 3, Figure 4).

**Table 3:** Cross-validation performance metrics on training set.

Models	PRE	SEN	SPE	ACC	F1	ROC-AUC	PR-AUC
LR	0.6659	0.8672	0.3486	0.6595	0.7533	0.7381	0.8004
KNN	0.7388	0.9389	0.5032	0.7644	0.8269	0.7488	0.8207
Adaboost	0.7368	0.9557	0.4890	0.7688	0.8321	0.8918	0.9311
XGBoost	0.7986	0.9357	0.6467	0.8200	0.8617	0.8989	0.9311
LightGBM	0.8225	0.8641	0.7208	0.8067	0.8428	0.8732	0.8939
AutoML	0.8922	0.9589	0.8265	0.9059	0.9243	0.9563	0.9559

### 3.4 Comparative assessment of predictive performance

**Figure 5:**
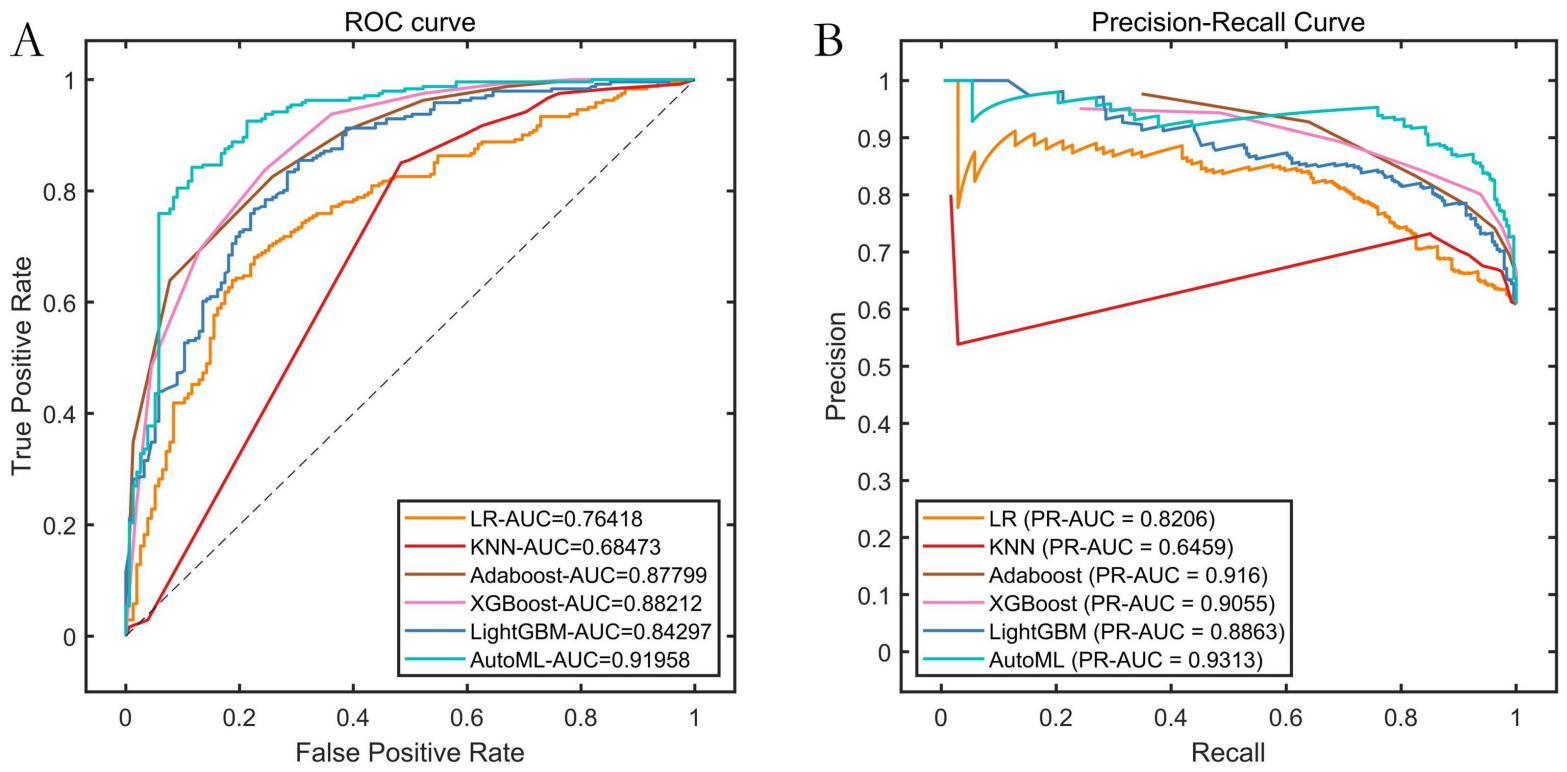
Cross-validation performance of the testing set. **(A)** ROC curve of testing set; **(B)** PR curve of testing set.

Results from the testing set confirm that AutoML persists with optimal predictive capability, achieving an F1-index of 0.8924, ROC-AUC of 0.9196, and PR-AUC of 0.9213, thereby indicating robust generalization capacity (Table 4, Figure 5).

**Table 4:** Cross-validation performance metrics on testing set.

Models	PRE	SEN	SPE	ACC	F1	ROC-AUC	PR-AUC
LR	0.7099	0.8631	0.4516	0.7020	0.7790	0.7642	0.8206
SVM	0.6950	0.9170	0.3742	0.7045	0.7907	0.6847	0.6459
Adaboost	0.7412	0.9627	0.4774	0.7727	0.8375	0.8780	0.9160
XGBoost	0.8014	0.9378	0.6387	0.8207	0.8642	0.8821	0.9055
LightGBM	0.7955	0.8714	0.6516	0.7854	0.8317	0.8430	0.8863
AutoML	0.8582	0.9295	0.7613	0.8636	0.8924	0.9196	0.9313

### 3.5 Analysis of application value

**Figure 6:**
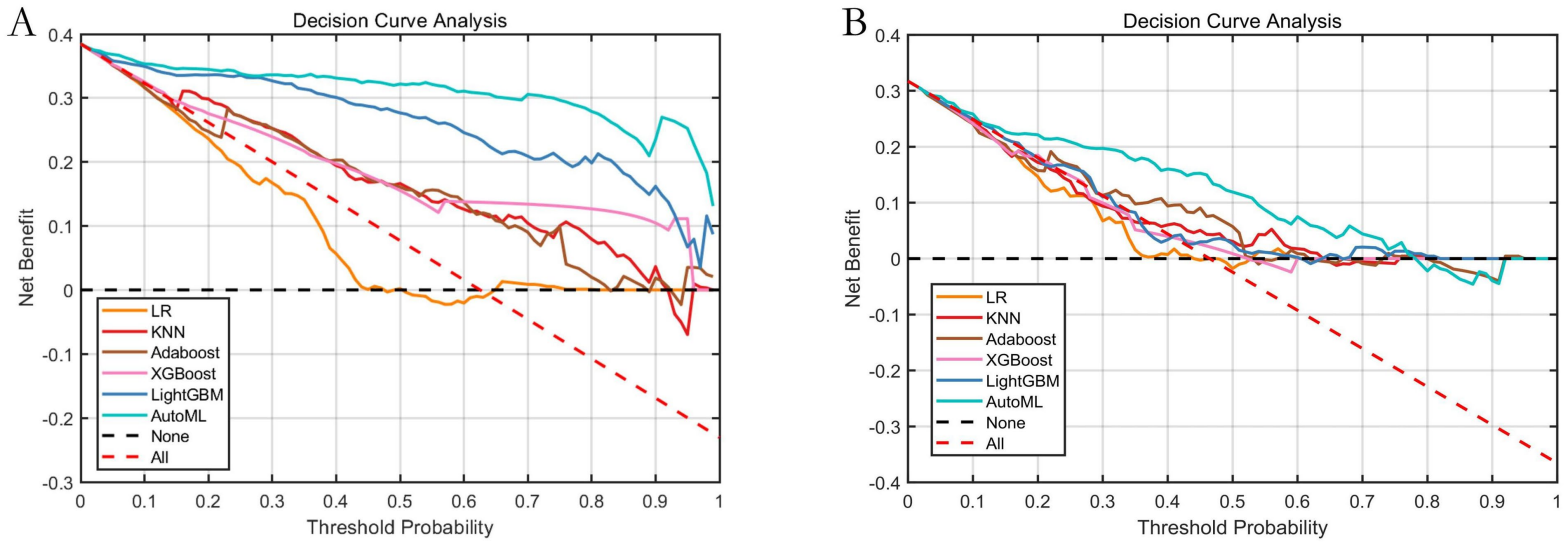
Decision curve analysis of the prediction model **(A)** training set and **(B)** testing set. Note: The Y-axis shows the net benefit, the realization represents the prediction model, the red dashed line represents the assumption that all patients develop delays, and the black dashed line represents the assumption that no patients develop delays.

The decision curve analysis pertaining to delayed healthcare access among Tibetan residents is illustrated in Figure 6. Results from the testing set decision curve indicate that employing AutoML for risk prognostication of healthcare-seeking delay confers superior clinical benefits compared to conventional methodologies across a risk threshold spectrum of 2 to 77%.

### 3.6 Interpretability analysis

(1) SHAP analysis.

**Figure 7:**
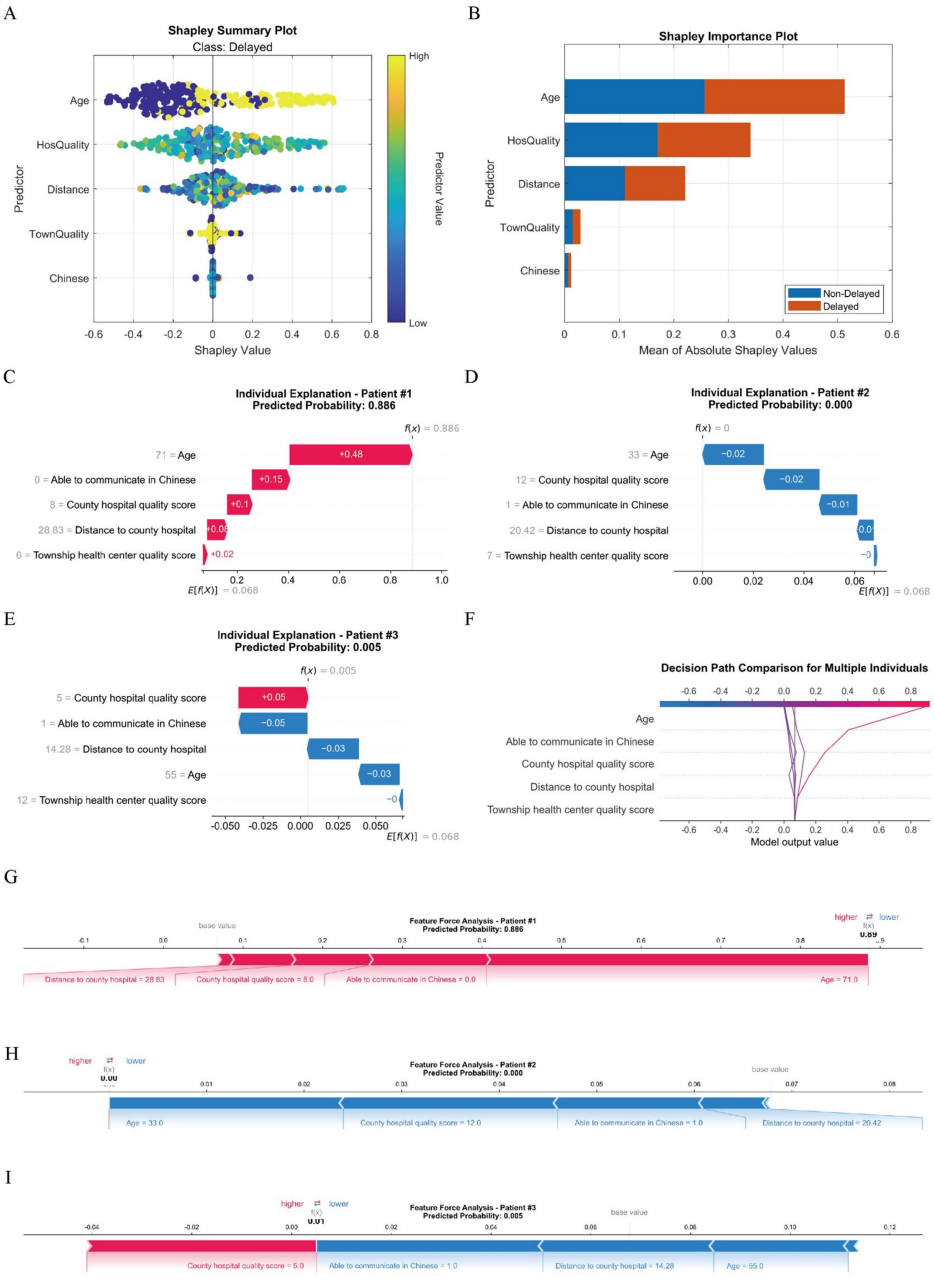
Machine learning interpretability analysis. **(A)** The Shapley summary plot comprehensively presents the overall impact patterns of various features on model predictions across all samples. Each point in the plot represents a feature and its SHAP value (i.e., the feature’s contribution to prediction) for a specific sample. The color of the points indicates the actual value magnitude of the feature (yellow for high values, blue for low values), while the distribution along the horizontal axis (SHAP values) reflects how feature values influence predictions (positive values increase predictions, negative values decrease them). This visualization allows intuitive identification of which features generally correlate with increases or decreases in predicted values, as well as trend relationships between feature influence and feature magnitudes; **(B)** The Shapley feature importance plot displays the overall importance ranking of each feature’s impact on model predictions in bar chart form. Feature importance is determined by calculating the mean absolute SHAP value for each feature across all samples, thereby measuring its average contribution to model output variations. Longer bars indicate greater influence of the feature in the model’s overall decision-making process, providing researchers with clear insight into the most critical factors driving predictions; **(C–E)** Waterfall plots illustrate the cumulative contribution process of each feature to individual patient predictions. The baseline value represents the model’s average prediction for all patients, while feature contributions show how each feature affects the final prediction (red indicating increased risk, blue indicating decreased risk). The sum of all feature contributions yields the final predicted value; **(F)** The decision path plot compares decision pathways across multiple patients, demonstrating how different feature combinations lead to varying prediction outcomes. The horizontal axis shows predicted probabilities, the vertical axis lists features, and the curved pathways trace decision routes from baseline values to final predictions; **(G–I)** Force plots visually demonstrate how each feature “pushes” predictions toward higher or lower risk directions. Red arrows indicate features pushing predictions toward higher risk, blue arrows indicate features pushing toward lower risk, with arrow length representing the magnitude of influence; HosQuality: County hospital quality score; Distance: Distance to county hospital; TownQuality: Township health center quality score; Chinese: Able to communicate in Chinese.

SHAP analysis outcomes (Figures 7A,B) reveal the following hierarchical feature importance: (1) Age; (2) County hospital quality score; (3) Distance to county hospital; (4) Community clinic quality score; (5) Able to communicate in Chinese. These five features represent the most important predictors identified through SHAP value ranking and were quantitatively validated as top contributors by three distinct methodologies: AutoML-SHAP, LightGBM’s intrinsic feature importance, and LASSO regression. Their selection reflects both statistically significant predictive dominance and cross-method consensus, with no absolute threshold applied. The selection criteria for these top five features were rigorously defined by (i) SHAP value magnitude ranking across all features, (ii) alignment with LightGBM’s native feature importance, and (iii) statistical significance in LASSO regression outcomes. While LASSO identified two additional variables (education level and self-rated symptoms), they were not prioritized by AutoML-SHAP analysis, confirming the consolidated importance of these five factors spanning patient-specific, geographical, and healthcare-resource dimensions.

Through the Waterfall Plot (Figures 7C–E), it can be clearly seen that for high-risk patients (such as Patient #1), the main risk drivers include advanced age, far distance to county hospital, low county hospital quality score, inability to speak Chinese, etc. For low-risk patients (such as Patient #2), their feature combination shows a low-risk pattern, such as younger age, closer distance, higher quality score, ability to speak Chinese, etc.; By comparing decision paths of patients with different risk levels (Figure 7F), systematic differences between high-risk and low-risk patients in feature combination can be found. The paths of high-risk patients obviously shift to the right, indicating multiple high-risk characteristics act together; Force Plot (Figures 7G–I) enables clinicians to quickly identify features with the greatest impact on prediction outcomes for specific patients. For example, in Patient #1’s Force Plot, “advanced age,” “far distance to county hospital,” and “inability to speak Chinese” can obviously be seen as main factors that push the prediction toward high risk.

**Figure 8:**
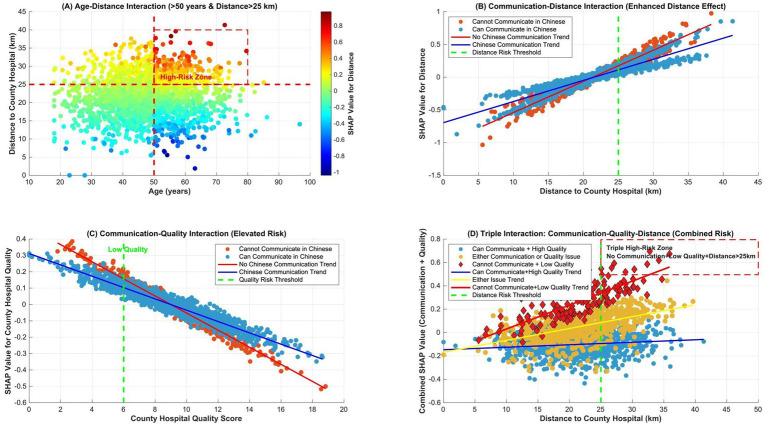
SHAP interaction analysis between key indicators.

SHAP interaction effects revealed that (Figure 8): (A) Dual high-risk group: Age >50 years and distance >25 km (the red area indicates high SHAP values). Risk was predominantly concentrated in older adult patients residing far from county-level hospitals. (B) Amplified distance effect: Among patients unable to communicate in Chinese, the influence of distance was amplified, indicating that language barriers exacerbate distance-related care delays. (C) Elevated risk: Patients unable to communicate in Chinese exhibited significantly heightened risk (red markers). This risk further increased when the quality score of county-level hospitals was <6. (D) Triple high-risk combination: The convergence of: inability to communicate in Chinese + low-quality county-level hospitals (quality score <6) + distance >25 km formed a triple high-risk cluster (red diamond markers), necessitating prioritized intervention.

(2) Comparison with Traditional Methods.

Table 5 presents a comparative analysis of feature screening results between the AutoML model (using SHAP values) and conventional LASSO regression. These findings demonstrate that: The five core predictive features identified via AutoML SHAP values show exceptional concordance (*κ* = 0.92) with LightGBM’s intrinsic feature importance ranking; Significant alignment exists between AutoML-selected features and LASSO regression outcomes, with fourfold consistency in clinically critical variables (Age, Distance to county hospital, County hospital quality score, Mandarin communication ability).

**Table 5:** Comparative feature selection: AutoML-SHAP vs. LASSO regression.

Characteristics	AutoML SHAP value	LightGBM importance	Lasso *β* value	Consistency
Age	0.27	6.78	1.15	√
County hospital quality score	0.17	5.85	−0.82	√
Distance to county hospital	0.11	4.52	0.68	√
Able to communicate in Chinese	0.02	3.38	−0.55	√
Community clinic quality score	0.01	2.08	−0.48	√
Education level	–	–	−0.35	×
Self-rated symptom severity	–	–	0.28	×

### 3.7 Clinical decision support system

**Figure 9:**
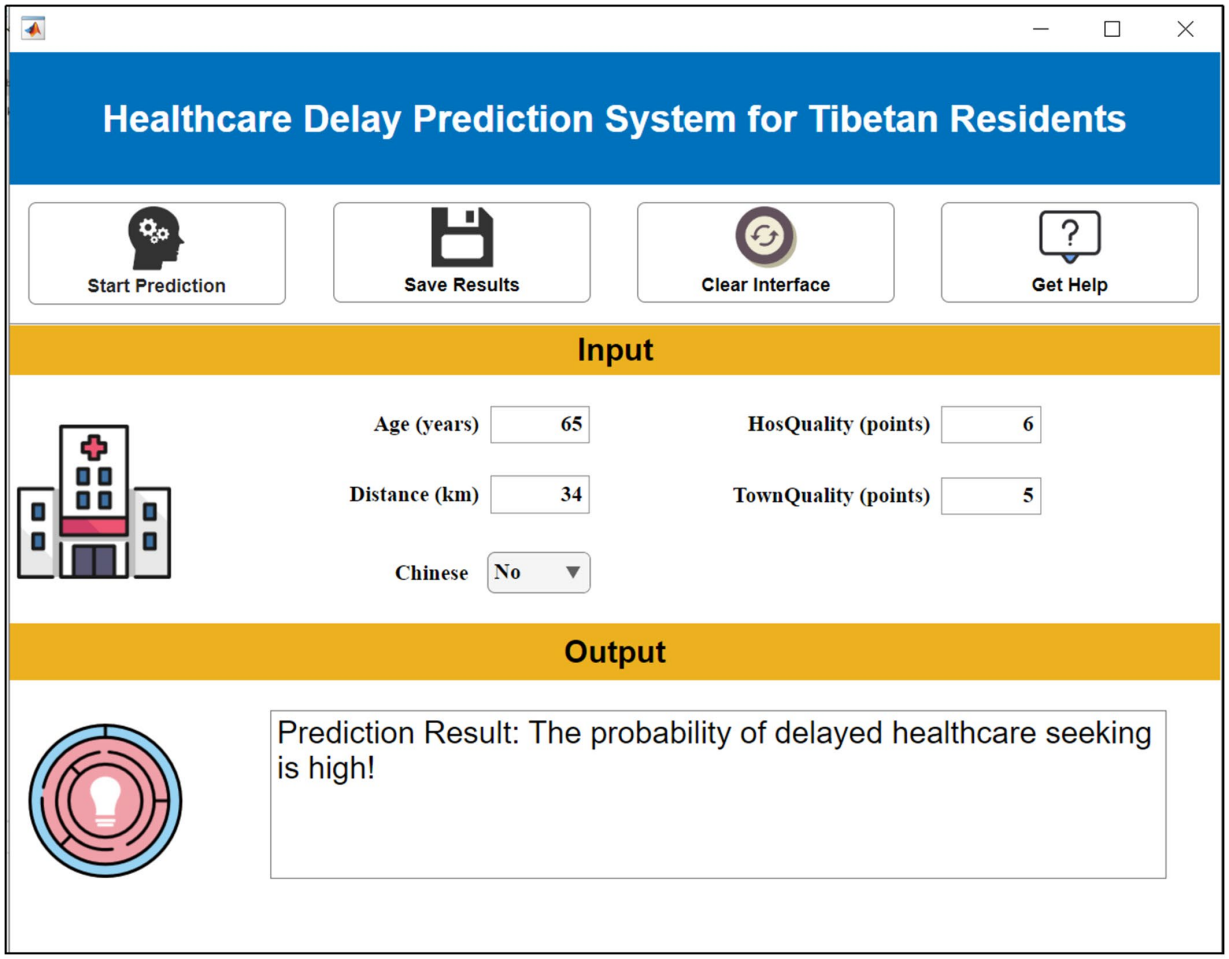
Demonstration of clinical decision support system.

The aforementioned research has successfully identified the key features affecting patient outcomes. However, in clinical practical applications, the variations of these features are complex and intricate, making it difficult to intuitively reveal patients’ prognostic risk. Existing artificial intelligence methods have high thresholds for promotion and application, requiring clinical staff to possess advanced programming skills and extensive literature knowledge, which makes them difficult to be widely promoted in hospitals. To solve this problem, this study innovatively constructed a practical visualization system; this system is built based on the selected key features and possesses the application advantages of intuitiveness, convenience, and practicality. During the application of the visualization system, users only need to input the specific values of five key features in the “Feature Input” column, and the system will automatically calculate blood transfusion requirements, as shown in Figure 9.

## 4 Discussion

In Tibetan regions, the phenomenon of healthcare-seeking delay among local populations arises from multifaceted determinants spanning natural constraints, cultural perceptions, and healthcare system dynamics (15, 16). Conventional prediction methods have struggled to concurrently satisfy requirements for accuracy and model transparency. To reconcile these dual needs, this study combines AutoML for predictive accuracy with post-hoc SHAP analysis for model interpretation.

Through analysis of 1,879 Tibetan residents (with a healthcare-seeking delay incidence of 41.99%), this study employed the IETO-AutoML framework to identify five pivotal determinants: age, county hospital quality score, distance to county hospital, Able to communicate in Chinese, and community clinic quality score. These factors intricately intertwine, creating a multidimensional delay mechanism. Age functions dually: physiological decline diminishes symptom awareness and mobility, while deeply rooted cultural beliefs—such as attributing illness to “karmic retribution” and prioritizing traditional ritual countermeasures—prolong the initial intervention window (17). Heterogeneous hospital quality drives divergent health-seeking behaviors: high-scoring facilities attract patients despite geographical barriers through advanced resources, whereas low-tier institutions induce “observational delay,” characterized by deferred referrals or protracted decision-making (18). Distance manifests not linearly but as exponentially escalating risks, compounded by primitive transportation, hypoxic high-altitude terrain, and prohibitive costs—collectively steering residents toward folk remedies while forfeiting the golden treatment window (19). Able to communicate in Chinese transcends mere communication barriers, evolving into systemic trust deficits: some patients decline essential care or await family interpreters, exacerbating health information asymmetry (20). Concurrently, community clinic quality modulates “proximity-based care” viability: high-quality clinics streamline decisions, whereas substandard facilities force “hospital-skipping behavior,” escalating temporal and logistical burdens—particularly during seasonal mountain blockades (21). These variables operate not only independently but also interact systemically, impairing healthcare decision-making, resource accessibility, and timely intervention. Multidimensional strategies—including culturally integrated approaches, spatial interventions, and economic support—are imperative to alleviate the aggregate delay burden.

The SHAP interaction analysis reveals critical synergies between risk factors that extend beyond main effect interpretations. Most notably, we identified a triple high-risk cluster marked by the convergence of language barriers, substandard county hospital infrastructure (quality score <6), and travel distances exceeding 25 km. This configuration manifests as exponentially amplified risk when older adult patients in high-altitude regions experience compounding geographical isolation—where primitive transportation networks and seasonal weather blockades transform distance into nonlinear care barriers. The analysis further demonstrates how language limitations paradoxically magnify distance effects: non-Chinese speaking patients experience pronounced delays even within moderate travel radii, reflecting systemic trust deficits and care-seeking hesitations due to informational asymmetry. These findings align with the hierarchical feature importance identified in our model while revealing crucial interdependencies undetectable through conventional statistical methods. The synergistic risk patterns suggest that isolated infrastructure improvements may yield limited benefit without concurrent strategies addressing linguistic-cultural barriers in remote Tibetan communities. Future interventions should prioritize integrated solutions for these high-risk clusters, such as deploying mobile clinics with multilingual staff to mountainous regions during winter seasons, coupled with telemedicine systems bridging quality disparities in county-level facilities.

This research delivers dual value: for clinicians, the CDSS enables point-of-care risk stratification using five intuitive inputs (age, language, distances, facility ratings) to prioritize high-risk groups like older adult non-Chinese speakers. Public health administrators gain operational insights from SHAP-identified risk clusters (language barriers + infrastructure gaps + remoteness) to optimize resource allocation, such as deploying mobile clinics to remote villages during seasonal disruptions. For data scientists, it provides a transparent AutoML framework that harmonizes predictive power with clinical interpretability in minority health contexts.

Further findings demonstrate that IETO exhibited outstanding performance across the majority of test functions, with significantly enhanced stability compared to the original ETO and other benchmark algorithms. Through its optimized framework, the model autonomously selected LightGBM as the optimal base learner. Cross-validation against five traditional models confirmed IETO-AutoML’s superior comprehensive performance, while test-set results verified AutoML’s optimal predictive capability—indicating greater clinical utility than conventional methods for forecasting healthcare delays in Tibetan regions. The advantage stems from AutoML’s autonomous capacity to integrate multi-source spatial data, sociocultural variables, and healthcare facility geolocation through automated feature engineering, thereby mitigating data bias risks. This automation overcomes limitations of traditional approaches requiring manual integration of complex, multidimensional data—a process prone to operational errors and parametric misconfigurations compromising accuracy. Empirical validation confirms AutoML’s superiority for imbalanced, multimodal data integration tasks (22, 23). For algorithmic selection, AutoML systematically evaluates diverse machine learning methodologies, utilizing validation techniques to identify context-optimal architectures. Conversely, traditional modeling often relies on subjective algorithmic choices vulnerable to parametric misconfiguration and predictive bias (24). Leveraging AutoML’s interpretability features, health institutions could scientifically reconfigure resource distribution or design culturally aligned services. Traditional models lack such precision management due to limited explainability. Additionally, when confronting evolving medical conditions, AutoML’s automated modeling pipeline—encompassing data cleansing and algorithmic training—reduces development cycles from weeks to hours. This allows medical teams to concentrate on intervention strategies, while conventional methods remain labor-intensive across all phases (25, 26).

This study has certain limitations, primarily concerning the generalizability of data samples and the scope of model applicability. First, data collection relies heavily on face-to-face questionnaire interviews, which, despite stringent quality controls like multidimensional safeguarding protocols and instrument reliability verification (Cronbach *α* > 0.8), are confined to Tibetan residents within a regionally confined sampling frame (Ya’an City, Sichuan Province), limiting result transferability to other Tibetan-populated areas such as the Tibet Autonomous Region or Qinghai. We are actively initiating multi-site validation across Tibetan Autonomous Region and Qinghai Province to assess model generalizability, with results forthcoming in future publications. Additionally, while critical factors such as language barriers and healthcare distances are included, the survey design omits comprehensive consideration of cultural beliefs or abrupt contextual variables, potentially causing the predictive model to overlook critical variable interactions. Second, although the AutoML-based framework offers efficiency, the feature importance analysis via SHAP-derived weights may introduce parameter biases under high heterogeneity, as demonstrated by insufficient sensitivity analyses of specific conditions in hypoxic environments. Future research should focus on expanding data dimensions and refining methodologies, specifically extending recruitment frameworks to include broader geographical representation across key Tibetan regions to enhance cross-regional validation and generalizability. Concurrently, advancing practical implementations, such as optimizing existing clinical decision support systems for mobile deployment or validating their feasibility in other chronic disease predictions, will enhance precision interventions. It should be specifically noted that this study utilizes AutoML to efficiently search for optimal configurations from a family of models inherently possessing strong interpretability potential. Although this search did not directly target interpretability metrics as objectives, the high-performance LightGBM model selected through this process exhibits native compatibility and computational efficiency with post-hoc explanation tools like SHAP due to its tree-based architecture; this establishes a foundation for subsequent in-depth and reliable interpretability analysis. Future work will explore directly incorporating interpretability constraints into AutoML objectives.

## 5 Summary

This study, employing empirical analysis of 1,879 Tibetan residents (healthcare-seeking delay incidence: 41.99%), innovatively established an IETO-AutoML-based predictive framework that successfully identified five core determinants. SHAP interpretability analysis validated the hierarchical significance of these variables, while automated machine learning simultaneously overcame dual limitations of conventional approaches—demonstrating substantial efficiency advantages in both feature selection and hyperparameter optimization. This advancement provides data-driven decision support for deploying precision interventions including dynamic deployment of bilingual medical teams, strategic mobile clinic planning, and customized transport subsidies. Collectively, these insights contribute meaningfully to alleviating disparities in equitable public health service accessibility across Tibetan regions.
